# An Apology for the Nerves; or Their Influence and Importance in Health and Disease

**Published:** 1845-01

**Authors:** 


Art. II.—*An Apology for the Nerves; or their Influence and Importance on Health and Disease.* By Sir George Lefevre, m.d. &c.—*London*, 1844. 8vo, pp. 364.

In laying down Sir George Lefevre's book we could not help exclaiming with Prince Henry, in Sir George's own vein—"We could have better spared a better man." Like the author's 'Life of a Physician,' the present book is clever and amusing, but we cannot say, that it is a particularly good medical book. Sir George is somewhat flighty and discursive, and is almost too humorous for a grave matter-of-fact writer on physic;—but we have been too much tickled with some of his narratives to be able to exercise the vocation of the critic in the wonted cool and crabbed style. We shall, therefore, let him pass scot-free this time; but we give him fair warning that his next book, if professional, shall be dealt with as strictly as if he were the dullest of doctors. But if we were disposed to do so, our present limits would not allow us to dwell on either the merits or demerits of the present volume. We must content ourselves with giving the reader a brief note of its principal contents, assuring him, at the same time, that though he may not derive a vast deal of novel information from it, he cannot fail to be both interested and amused by its perusal.

The work is divided into twelve parts and an appendix. Part first is devoted to a consideration of the brain and nerves, respiration and animal heat. Sir George Lefevre here labours to show the primordial rank and importance of nerve over muscle and blood, which last he admits to be "collaborators" with the former in all the functions of life, but not "coequals" with it. (p. 10.)

As regards respiration, we are informed (p. 17,) that its uses are the "purification of the blood and the generation of animal heat." In the function of respiration, "assumed" by some to be "wholly chemical," we, in the author's opinion, "recognize" the nervous power in the "voluntary" action of throwing the blood into the chemical laboratory." (p. 17.) Though in a chemical point of view, "the whole phenomenon of animal heat is resolvable into the different capacities with which certain forms of matter are endowed with for caloric," or to a process of combustion, similar to that of charcoal, out of the body, and by which the carbon of the blood, uniting with oxygen, and being converted into carbonic acid, gives out heat, yet observes the author, (p. 18,) "a host of evidence proves, that, however this process may be effected, .... it is the brain which has the supremacy in the function; for were it merely chemical, the same conjunction of matters would produce the same effects, which does not prove to be the case." And then he refers to Sir B. Brodie's experiments on artificial respiration.

In perusing the following parts on the blood, muscular motion, circulation, nutrition, and secretion, the reader will be much interested, as well as by the notices which follow on sympathy, phrenology, mesmerism, sleep and dreams.

In part V, and up to XI inclusive, a variety of matters, physiological, pathological, and therapeutic, are brought under notice, and made the subject of popular illustration. Among these, we may instance the five senses, voice and speech, the influence of the blood upon the nerves, nervous complaints, headaches, epilepsy, hysteria, palsy, catalepsy, hydrophobia, &c. &c., fevers, Moldavian fevers, sciatica, iritis, earache, homœopathy, instinct and reason, memory, &c. Most of the pathological and therapeutical notices are illustrated by some case or cases, most graphically detailed,—and proving that the author is very capable of writing a much better book than he has now favoured us with.

In part XII, which is entitled "German therapeutics—Some peculiarities of German practice," (p. 271)—we meet with some interesting particulars, one or two of which we shall notice. "The Germans," Sir George informs us, "boast of a simplicity of prescription, and have a horror of contrarieties, carrying this to a ludicrous nicety, and an unmeaning orthodoxy." Anything stronger than distilled water is regarded as not inert. At page 281, what is called the grape cure is described. It is employed in "functional nervous affections" in persons of higher rank, which have resisted ordinary methods of treatment. A lady of quality migrates into the country, turns a poor peasant out of his hut, (amply recompensing him, however,) endures its filth, sleeps on the peasant's crib, and "uses the grape alone for meat, the grape for drink." This regimen continues for three weeks or a month.

Muriate of ammonia is employed in cases of "serous and mucous congestion," (p. 287,) and along with tartarized antimony in pleuritic affections. Nitrate of soda is considered by the Germans as more antiphlogistic than nitrate of potash. Phosphoric acid is employed in all those cases in which British practitioners use sulphuric and muriatic (hydrochloric) acid. An etherial tincture of digitalis seems to have some advantages over the one employed by us. Indigo is a popular remedy with the German physicians in cases of epilepsy, hysteria, and in all convulsive diseases of children. Extractum graminis (triticum repens) is considered "a mild tonic, and deobstruent in the convalescence of fever." It is greatly used, as well as the extract of taraxacum. Ox-gall and the oleum jecoris aselli appear to be a good deal prescribed.

In the appendix to the work, which occupies nearly a fourth part of the volume, and for the dissociation of which from the rest of the work we can perceive no cause, several very odd cases are narrated. One of the oddest is given at p. 323, under the head "Credat Judæus," extracted from a work in the Romanzoff library, at St. Petersburg: A woman was brought to bed of a girl, who, in its turn, eight days after its birth, was taken with labour-pains, and gave to the world an "embryo" as long as the middle-finger, which, as it bore the human form, was baptized, but died (alas!) along with its mother, the day after.

Under the head, "Cæsarean operation," Sir George mentions the case of a woman at St. Petersburg, who had *twice*, and successfully, undergone the operation.

In a notice of Plica Polonica, Sir George informs us that as he has witnessed the disease in five opulent families, the pathogenical theory which derives it *wholly* from filth, must be rejected. It appears that the lower orders in Poland believe that it relieves many other diseases, pains, &c., and actually take means, by keeping the head long wrapt up in flannel, to induce the plica artificially. Sir George says little or nothing about the treatment.

